# Testing impacts of global blur profiles using a multiscale vision simulator

**DOI:** 10.1016/j.heliyon.2020.e04153

**Published:** 2020-07-23

**Authors:** E. De Lestrange-Anginieur, C.S. Kee

**Affiliations:** a School of Optometry, Hong Kong Polytechnic University, Hong Kong; b Interdisciplinary Division of Biomedical Engineering, Hong Kong Polytechnic University, Hong Kong SAR, China

**Keywords:** Biomedical engineering, Optics, Blur, Spatial interaction, Isoplanaticity, Visual field, Observer method

## Abstract

Although it is possible to specify the impact of blur at a specific retinal location, a lack of understanding exists regarding how the inhomogeneous blur distribution across the retina (i.e., global blur) affects the quality of an optical correction at a specific retinal location. To elucidate this issue, a multiscale visual simulator combining the projection of a controllable high-resolution stimulus and an ocular monitoring system was constructed to simultaneously simulate foveal and extrafoveal blurs. To define the range and capability of a wide-angle stimulation, an optimal working pupil was evaluated by optical ray-tracing via a Monte Carlo simulation, including optical variations corresponding to fixational eye movements. To investigate the impacts of global blur on the perception of discrete regions of the visual field, the bothersome blur threshold from five subjects was measured through this novel system using a collection of zonal blurs (annuli image projected sequentially at discrete retinal regions), and these impacts were compared with those using a spatially-varying blur (continuum of simultaneously projected zonal blurs of varying strengths, simulating retinal blur variations). Our results show that the zonal blur threshold does not entirely predict the global blur threshold, having a tendency to overestimate blur the threshold. It was concluded that, in addition to the amount of defocus present at a defined retinal location, the perception of individual defocused retinal regions can be affected by global blur. Given that blur tolerance can affect the perception of optically induced blurs, the findings provide useful implications for designing new optical correction.

## 1 Introduction

Investigation of the benefits of a total correction of blur over the central area of vision (e.g. 20°) constitute a challenge in the optical control of retinal images due to the inhomogeneity of the retinal image quality. Image quality is usually considered invariant with eccentricity within approximately 2° [1], an area called the isoplanatic patch. Within this patch, a single deformable mirror, which uniformly corrects the entire field of view, can be used to project a high-resolution image onto the retina [2]. Beyond this patch, however, ocular aberrations can vary strongly with field angle and angular positions [3], depending on the retinal shape and the individual optics. Correction of aberrations over a larger retinal patch becomes thus more challenging, requiring the combination of several deformable mirrors and wavefront sensors [4]. To date, there is no visual simulator capable of simultaneously stimulating distant regions across the macula, while controlling ocular aberrations. Because of the complex spatial distribution of aberrations, theoretically there are multiple solutions for correcting the overall eye blur if the optical correction is extended over a large visual field. In ophthalmic practice, however, only an isolated region of the visual field is corrected by optical corrections. The prioritization of foveal correction can often lead to arbitrary optical modifications [5] in the peripheral visual field. One example is the unwanted peripheral blurs arising from progressive addition lens (PAL) design [6].

The optical control of ocular aberration is not only problematic when enlarging the retinal extent of correction (i.e., simultaneously correcting different retinal locations), but also if simply simulating retinal blurs at different spatial locations simultaneously. While adaptive technology can increase the level of control of a visual correction, it usually shrinks the extent of the visual stimulation. This has encouraged utilization of the “source method” [7] when manipulating blur over space [8] or time [9]. In fact, most visual simulators intended to test peripheral blur or control blur are designed for a small visual angle that does not allow simultaneous testing of the fovea and parafoveal regions, with a restriction on sequential testing [10, 11]. As a result, it is unknown to what extent visual perception is affected simultaneously by global blur at a given retinal location. Interestingly, both animal models [12] and human studies [13] show evidence of an impact of peripheral defocus on eye growth, suggesting a potential interaction over time between peripheral and foveal blurs. Although this process may not involve the brain [14, 15], to date, few studies have examined this interaction at the perceptual level [16, 17].

This study aimed to investigate whether stimuli at different retinal locations are affected by the spatially varying blur of the eye. Our hypothesis is that peripheral blurs can alter blur perception of the retinal image at different locations. Considering the residual peripheral aberration induced by optical corrections (e.g., IOLs, multifocal contact lenses), such contextual modulation may be important to predict blur perception, when correcting a local region of the retina, typically, the foveal or the preferred retinal locus. To test this hypothesis, a multiscale vision simulator (MVS) with an extended isoplanatic patch that minimizes the inhomogeneity of image quality along angular and radial positions of the visual field was developed, which could reduce the effect of the aberrations of the eye on individual retinal images allowing testing of the eye independent of the individual ocular aberrations, rather than to correct aberration. This was achieved by the projection of high-resolution (4K) images, which were viewed with an optical artificial pupil, the size of which was adjusted to provide a wide-angle, macular diffraction limited image. Using the MVS, natural ocular aberrations were minimized, and an image-processing blur was imposed on the eye allowing measurement of blur perception independent of individual ocular aberrations. The results show that the bothersome blur threshold at a given location could be affected by peripheral blurs. Thus, it is plausible that manipulation of peripheral blurs, purposedly or not, a refractive correction can vary blur perception at the location targeted by a correction (e.g., at the fovea), although further studies are required to elucidate the extent of those blur interactions and whether they can affect an optimal correction.

## 2 Methods

### 2.1 Design and evaluation of the apparatus

**Figure 1:**
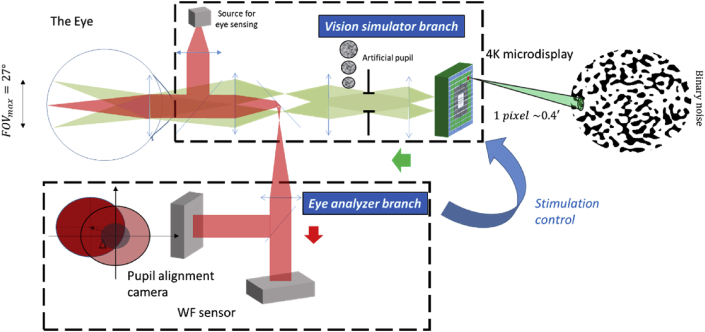
Generalized one-dimensional schematic of the newly developed MVS showing both the projection of the multiscale image (in green) and the illumination, as well as the light detection pathway (in red) for ocular analysis and monitoring eye movement during the test. The monochromatic light path was controlled by using an adjustable artificial pupil, tuned to the visual angle selected. A key asset of the system is the high-resolution image projection that can stimulate the varying spatial scales of retinal cells present along the macula, such as foveal photoreceptors of a few microns width.

This system allows a simultaneous stimulation of blurs in foveal and extrafoveal locations across the varying spatial scales of retinal cells present along the macula. As shown in Figure 1, the MVS is comprised of two branches: a psychophysical branch; and an analyzing branch. An illumination arm projected an infrared light beampupildiameter:1mm,λ=840nm±50nm) onto the eye using a superluminescent diode coupled to an optical fiber (Superlum, Ireland). Light reflected from the eye was directed to the analyzing branch comprising a wavefront sensor (HASO32, Imagine Eyes, France, 32 x 40 lenslets) and a pupil alignment control system. Image quality was monitored in real time (pupil size D = 1.35 mm, 15 Hz) by recording the on-axis wavefront errors of the subjects, and quantified by the root mean squared (RMS) wavefront errors. Throughout the test, the position of the pupil was adjusted by the experimenter by means of a pupil alignment camera using a three-dimensional translational stage fastened onto a chin-rest. A thin black ring (radius: 1.5°; thickness: 2 arcmin) was concomitantly displayed in the center of the display to assist subjects to maintain stable fixation without obstructing the central foveal zone. While the pupil position was monitored over time, a monochromatic green image (λ=550nm±20nm) generated by a light projector (DLA-X700R, JVC Inc.) was projected onto the eye via a long focal length lens-based telescope (retinal magnification: 0.5) to provide a diffraction-limited retinal image over a large visual angle (see section **B**). Equipped with JVC's e-shift technology [18], the projection unit consists of three liquid crystal on silicon LCOS microdisplays (diagonal size: 0.7′) covering a full visual angle of approximately 27° × 15°, which together produced a pixel stimulus that was approximately the size of a foveal cone (i.e., 0.4arcmin°). Light uniformity was also tested using a CCD camera placed at the pupil and at the retina of an artificial eye. In order to minimize the aberrations of the eye plus system, an adjustable artificial pupil is conjugated to the eye pupil, which can be flexibly tuned to the requirements of the stimulation (i.e., viewing angle, resolution, and eye movements). To calibrate the system, light emitted by the projector at different eccentricities was measured by a wavefront sensor placed at the pupil plane of the eye.

### 2.2 Methodology for estimation of artificial pupil size in wide-angle testing

With free viewing display, the simulation of a retinal stimulus with different blurs is limited by variation of aberrations occurring over time, as well as the large inhomogeneities of blur across eccentricities and angular positions of the retina. To minimize these inhomogeneities and to achieve a wide-angle diffraction-limited image, a simple approach was employed to reduce the pupil diameter of the imagery via an artificial pupil. In spite of the large reduction of ocular aberrations, this approach could still be significantly affected by eye movements (as described in the simulation below), but the appropriate choice of an angular window of simulation is often unspecified in published reports. Thus, it was attempted to determine the optimal range of working pupil required to maintain a wide-angle diffraction-limited image under fixational eye movements. Fixational eye movements are usually split into three categories [19], describing distinct motion dynamics: drifts, slow but large amplitude motions, which shift the position of the stimulus fixated onto the retina; microsaccades, a rapid, jerk-like movement (>2–3 Hz), which corrects displacement due to drifts by displacing the image from a dozen of foveal photoreceptors; and tremor, a very fast (~90Hz) but small amplitude (about diameter of foveal cone)movement. These movements create displacement of both the retinal images and pupil positions, which can modify on- and off-axis ocular aberrations.

**Figure 2:**
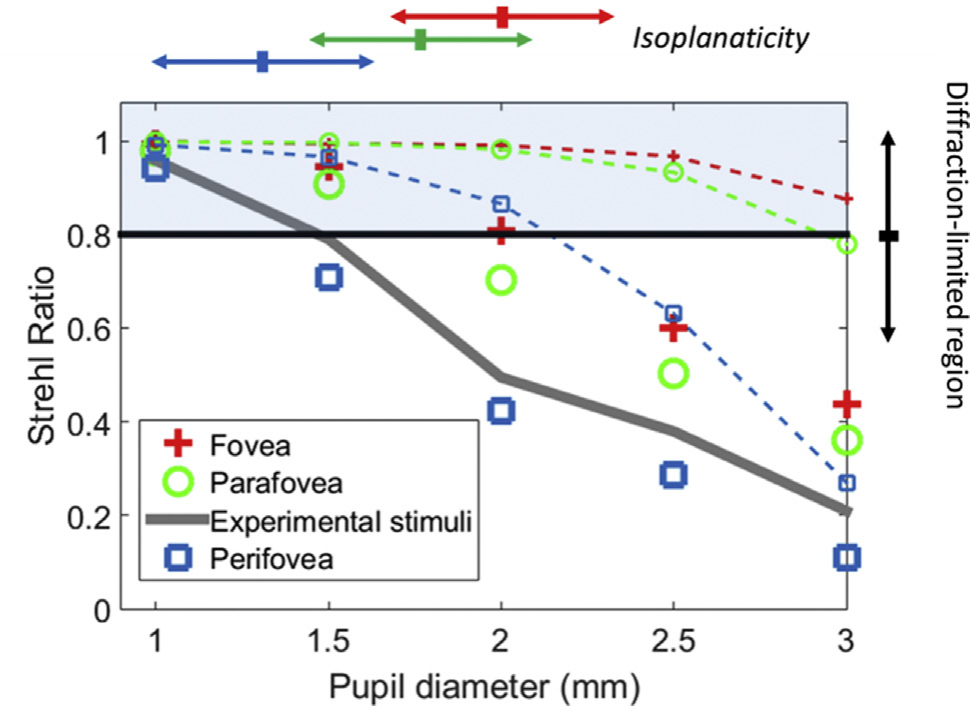
Simulated retinal image quality quantified by the Strehl ratio as a function of pupil diameter for various retinal extents. Dashed lines with small symbols correspond to the absence of fixational eye movements. In this condition, irrespective of pupil size, the diffraction-limit was achieved. The diffraction limit is indicated by the shaded area, which corresponds to a Strehl ratio of S > 0.8. Large symbols correspond to the worst optical quality over the entire selected field predicted by a Monte-Carlo simulation when introducing small fixational eye movements. In this condition, image quality was severely reduced for larger pupils. Distinct regions in image quality appeared: (1) an isoplanatic patch covering regions within the parafovea where the diffraction-limit was maintained up to a pupil diameter of 1.5 mm; and (2) an isoplanatic patch covering regions within the perifovea, up to a pupil diameter of 1.35 mm. The double-headed arrows on the top right side point the range of pupil size for which the variation of image quality was negligible and not. The arrow color (red, green, blue) indicates the associated retinal extent (fovea, parafovea, perifovea).

The optical design software Zemax was utilized to build a wide-field average eye model and perform fixational eye movement analysis. Numerous schematic eye models exist to predict the ocular aberrations in the visual field of healthy eyes. In this study, the Navarro eye model [20] was selected because it provides a basic and accurate anatomical description of the human eye to describe the field aberrations of an average eye. The Navarro eye model comprises four conic optical surfaces plus a spherical image surface describing the retina, which are separated by four refractive indices (including the cornea, aqueous, lens, vitreous). The wavelength was set at 550 nm and considered five retinal eccentricities (0, 2.5°, 4.25°, 9.25°), with eight angular field points per retinal eccentricity. In order to examine the isoplanaticity at different pupil sizes, ray tracing was performed for three retinal extents (radius: 0–2.5°), (radius: 0°–4.25°) and (radius: 0°–9.25°), delineated by foveal (2.5°), parafoveal (4.25°), and perifoveal outer edge (9.25°), respectively. Firstly, the ideal image quality achievable (in absence of eye misalignment) was calculated for various pupil diameters, ranging from 3mm to 1mm, by 0.5mm steps. The Strehl ratio of the worst point spread function (PSF) field over the entire selected field was used to determine whether the wide-angle image could be considered as diffraction-limited (peak PSF ≥0.8) over the full retinal extent tested. The results in Figure 2 show that, over this range of pupil diameter, a wide-angle diffraction-limited image could be achieved for all the tested pupil sizes, provided that the eye did not move. Nevertheless, when the eye fixates on a target, involuntary eye movements can cause the light rays to deviate from an ideal trajectory, bringing changes in the total aberrations, as well as foveal aberration.

Monte-Carlo analysis was performed to compute the change in optical quality due to the misalignment of the eye [21] caused by these eye movements. To simulate the eye movements likely to occur during the monitored visual simulation, a set of perturbations, including the tip/tilt of the eye, as well as radial decentering, were introduced into the wide-field eye model, randomly selected from a uniform number distribution. It was assumed that the amplitude of eye displacements would be smaller than 1.5mm (radial decentering: +/-0.75mm) and subjects’ fixation to remain centered within a ring of approximately 0.7° in diameter (tip/tilt: +/-0.35°). Although eye movements may vary significantly between subjects and time conditions, the value assumed was much smaller than the mean pupil displacements of 40 ± 10 μm previously reported for healthy fixating eyes in the absence of manual adjustment of the pupil [22]. A large set of 5,000 perturbations was simulated to cover the combinatorial perturbation susceptible to occur during the fixational eye movements assumed. After running the Monte-Carlo simulation, the worst-case scenario among the 5,000 eye model copies was extracted in order to estimate the largest optical/visual degradation that could occur under the eye displacements during visual simulation, and determine the pupil sizes required to maintain the limit of diffraction across all field positions. The RMS wavefront errors [23] was chosen as a metric to provide an objective comparison of image quality between the distinct visual conditions, since it is insensitive to the variations of neural filtering with pupil and field positions. It is important to note that, unlike on-axis, there is no consensus on what is the best metric for describing the effect of aberrations on wide-angle visual performance. The Monte Carlo analysis in Figure 2 shows that, while the anticipated diffraction-limited gain above a pupil diameter of 1.5 mm is contingent on small fixational eye movements, isoplanaticity is relatively well maintained at large visual angles via the use of smaller pupil size.

**Figure 3:**
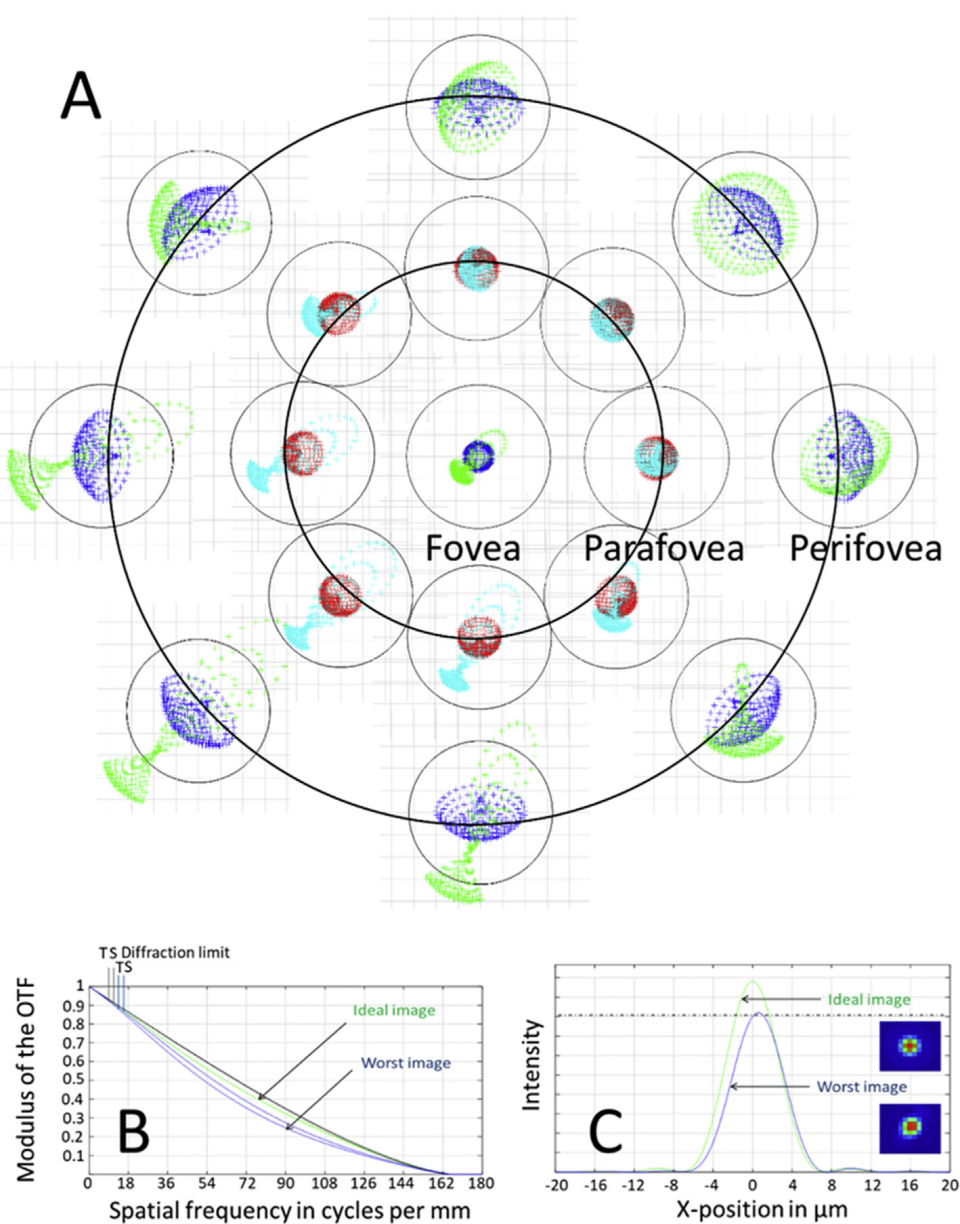
(A) Spot diagrams projected by the system at the retinal image plane of the eye across different retinal regions, highlighted by black isoeccentric rings, in the ideal (dark blue and red colors) and worst-case scenario (green and light blue colors) predicted by the Monte Carlo simulation. The black circles correspond to the first minimum of the Airy disk (radius: 6.5μm; D = 1.5 mm; λ = 550 nm). Note that, for a pupil diameter of 1.5 mm, image quality remained diffraction-limited over a large part of the macula. (B) Modulation transfer function and (C) point spread function of the ideal and worst-case scenario at the perifovea corresponding to the worst optical quality over the entire field for our selected pupil (D = 1.35 mm).

The spot diagrams of the retinal images over the visual field is shown in Figure 3A for both aligned and misaligned eyes. This observation indicates that pupil size adjustment not only constitutes a strategy to enhance image quality on axis, but also to achieve a diffraction limit over a wider spatial extent.

### 2.3 Optical performance using a pupil size of 1.35 mm

Based on this simulation, a visual angle of 15° was selected, and the artificial pupil set to a pupil diameter of 1.35 mm, which could allow testing of high resolution stimuli. As shown in Fig 3B and C, the highest resolvable feature was approximately 45 cycles/° (given the display sampling of three pixels per cycle), which is above the clarity of vision normally expected at 30cpd for 20/20 and largely beyond the range of contrast sensitivity of the human eye in extrafoveal location (eccentricity>1.5–2°), as well as, the spatial frequency for which contrast sensitivity is maximal at the foveal center [24, 25].

Importantly, for most visual tasks in real life settings, high spatial frequencies above 30cpd are not used, as predicted by the range of contrast sensitivity in the human eye in foveal and extrafoveal regions. Therefore, the limitation of this reduced pupil approach can be considered minimal for simulating the visual effect of blurring. In addition, the use of a small diameter pupil of 1.35mm effectively reduced the aberration of the optical system and the eye. The maximum optical change for the telescopic system was an approximate 20 nm wavefront error rms over the full field of view in both horizontal and vertical directions, which corresponds to λ/25. In a pilot measurement (sample N = 5), after objective refraction, we found a mean RMS wavefront error across subjects of 0.072±0.02μm, a value that is only slightly larger than the optical limit imposed by the Marechal criterion (lambda/14 = 0.06μm) for a diffraction-limited system. The temporal variation of image quality was small with a mean standard deviation of average RMS wavefront error of approximately 0.022±0.004μm, across subjects for the approximately 10-min time period of the visual test. The fluctuation in Zernike defocus coefficient was only 0.018±0.004μm. Most importantly, the image quality exhibited minor variations over the spatial positions of the display, with a mean standard deviation of average RMS wavefront error of approximately 0.010±0.005μm, across the central (0°) and four cardinal (7.5°) locations of the display. Optical degradation was, therefore, considered sufficiently small for a comparison of digital blur images.

### 2.4 Experiment

#### 2.4.1 Subjects

Five young trained subjects (20–35 years old) participated in the experiment. All observers were trained with psychophysical procedures and informed of the purpose of the experiments. The experimental procedures were approved by the Human Subjects Ethics Sub-committee of the university (HSEARS20170103001), and the research was conducted according to the principles expressed in the Declaration of Helsinki. Informed consent was obtained from each participant. The test was performed monocularly on the right eye. Given the small fluctuation of accommodation associated with targets monocularly viewed with a small pupil size and at far distance [26], the administration of cycloplegic drugs on subject eyes was dispensed with. In order to optimize image quality, defocus was corrected on -axis via a Badal lens by instructing the participant to subjectively report the clarity of a Landolt C target of approximately 20 arcmin viewed with the artificial pupil chosen for the test.

#### 2.4.2 Stimulus

**Figure 4:**
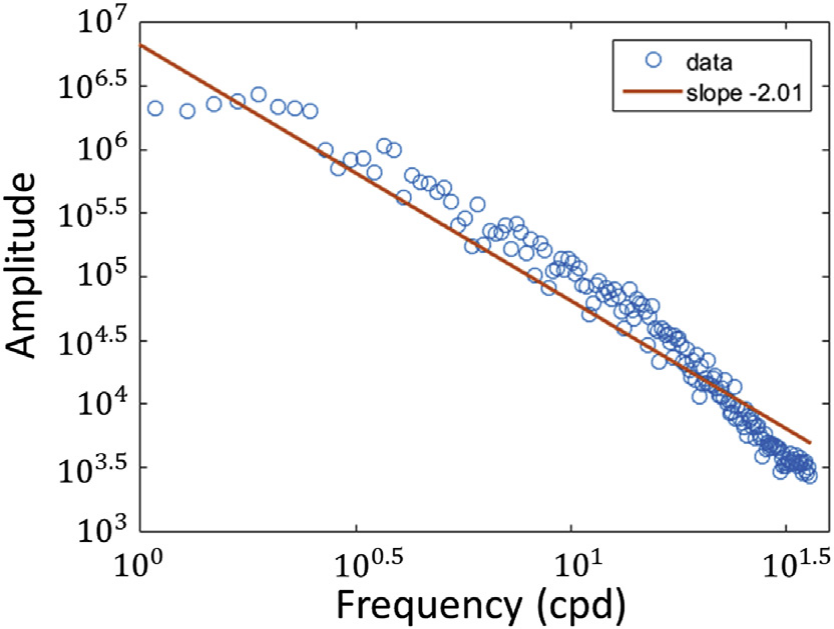
Amplitude spectrum of the M-scaled binary noise calculated along one direction. The straight line is obtained by fitting the log amplitude versus log frequency with linear regression. The amplitude spectrum falls roughly as “1/F” on log–log axes with a slope of -2.01.

**Figure 5:**
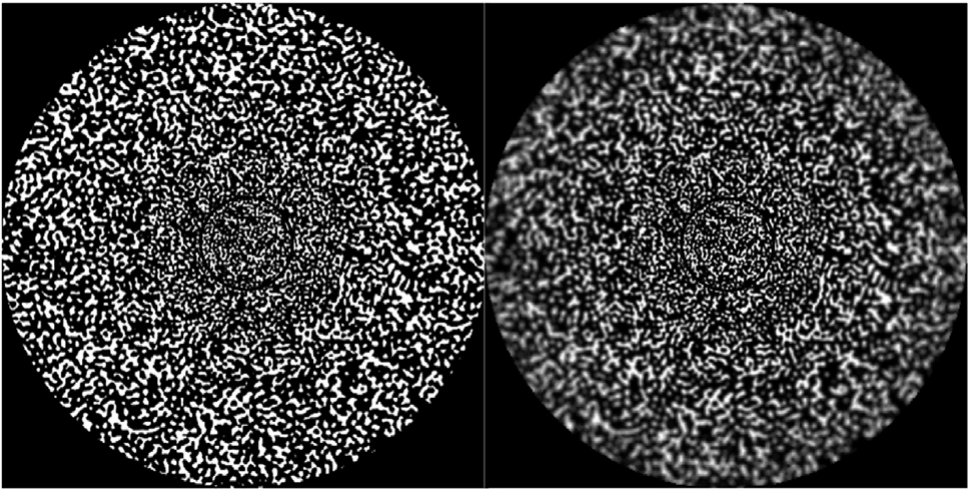
Original (left side) and spatially-varying blurred (right side) images of the M-scaled binary noise stimulus. Note that the binary noise exhibits sharp edges, readily apparent when zooming on the high resolution image (2160 × 2160), but which may be less visible in the reproduction. The spatially-varying blurred images is a superposition of a set of M = 20 concentric annular blurred images (width = 0.375°) obtained by convolution of the binary image with M PSFs of varying levels of Zernike defocus (from 0D to 5D, by step of 0.05D), computed for a monochromatic light of 550nm and a 2-mm pupil diameter. The radial change in Zernike defocus magnitude of the binary noise annuli followed a sigmoidal distribution that is determined by the slope of the sigmoid function and the defocus peak only.

The stimulus selected was a 4K binary noise having a sharp edge structure, as described in Figures 4 and 5. The full binary noises were depicted in color code, due to the difficulty in reproducing 4K blurred images (3840 x2160) in small print. The primary goal of the binary noise was to present a controllable stimulus that would broadly excite the photoreceptors and be free from the bias of natural images towards specific orientations, features, and regions. Previously used for assessing the visual effect of ocular aberration [27, 28, 29, 30], this stimulus is particularly useful for measuring small and subtle variations in blur. The binary noise stimulus was produced from a uniform noise distribution and filtered in the Fourier domain. The size and spatial scale of the stimulus were varied with eccentricity in order to roughly compensate for the change in the cortical magnification M of the retina in the visual cortex, using an approximate value of the human M [31]. This compensation produces a wide-angle stimulus with a more natural appearance because it minimizes variations in perceived sharpness across eccentricity [32].

#### 2.4.3 Visual tasks

To minimize variations of the defocused retinal images processed by the visual system, subjects were asked to fixate steadily on the center of the display throughout the test. Because the subjects were young and trained optometry subjects with previous experience in psychophysics experiments, it was assumed that subjects could maintain steady fixation, to avoid the need for a gaze-contingent stimulation, and so changes in visual fixation were not tracked. At the beginning of the experiment, subjects were informed that they would observe binary noise images with different levels of blur. The subjects were asked to report the level of blur that was unacceptable [33, 34, 35] by pressing the appropriate key. Some oral instructions were given to the subjects to highlight the criteria to be used: *Images with different levels of blur will appear on the screen. Whenever the alteration of the image clarity makes the image appear perceivably annoying or troublesome press the “arrow up” of the keyboard, otherwise press the “arrow down”.* Subjects were encouraged to base their judgment on the overall image, rather than just on regions near the fixation point.

#### 2.4.4 Blur generation

4K blurred images were computationally generated by convolution of the two-dimensional luminance profile of the binary noise and an isoplanatic point spread function (PSF) [36]:

$$PSF\left(x,y\right)={\left(F\left[g\left(u,v\right)\right]\right)}^{2}$$

$$g\left(u,v\right)=A\left(u,v\right)\text{exp}\left[\frac{i2\pi }{\lambda }W\left(u,v\right)\right]$$

where λ is the wavelength, *F* denotes the Fourier transform, A(u,v) describes the pupil function, W(u,v) refers to the wavefront aberration, which is described by a set of Zernike polynomials [37]. In this study, the wavefront aberration was calculated from the Zernike defocus polynomials $Z_{2}^{\text{0}}$ only, as:

$$Wdef\left(u,v\right)=c_{2}^{\text{0}}Z_{2}^{\text{0}}\left(u,v\right)$$

The amount of defocus $c_{2}^{\text{0}}$ used in the experiment, is expressed as [38]:

$$c_{2}^{\text{0}}=\frac{Mr^{2}}{4\sqrt{3}}$$

where *M* is the spherical equivalent in diopters and *r* the radius of the simulated pupil.

The PSF was calculated for a 2 mm diameter pupil, assuming monochromatic aberrations only with a wavelength λ = 550-nm. The pupil size was chosen to be sufficiently large to simulate the effect of blur, and minimize the computed size of the PSF matrix for the 15° viewing angle. For computational speed purposes, a set of defocused PSFs covering a range from 0 to 5D, by step of 0.05D, was used to pre-compute each image blur and load it just before the start of each visual test. Each blurred image was sorted into a set of annular isoplanatic blur images by applying annular masks at each eccentricity. The width of the annular masks was chosen to be smaller than the isoplanatic patch of the human eye, estimated to be about 2° [1]. Blur image variations were generated by the superimposition of the discrete annular isoplanatic blur images associated with each blur level, which provided a simple and flexible solution for blur manipulations and fitting the time constraints of visual testing. A GeForce GTX980 graphics card driving the projector was used to display the stimuli generated on a PC computer using PsychToolbox routines in MatLab software [39]. The binary stimulus (peak luminance: 15 cd/$m^{2}$; frame rate: 24 Hz; gamma: 2.2) was presented for a duration of 500 ms and interleaved by a green background for approximately 1.5 s.

#### 2.4.5 Experimental testing

**Figure 6:**
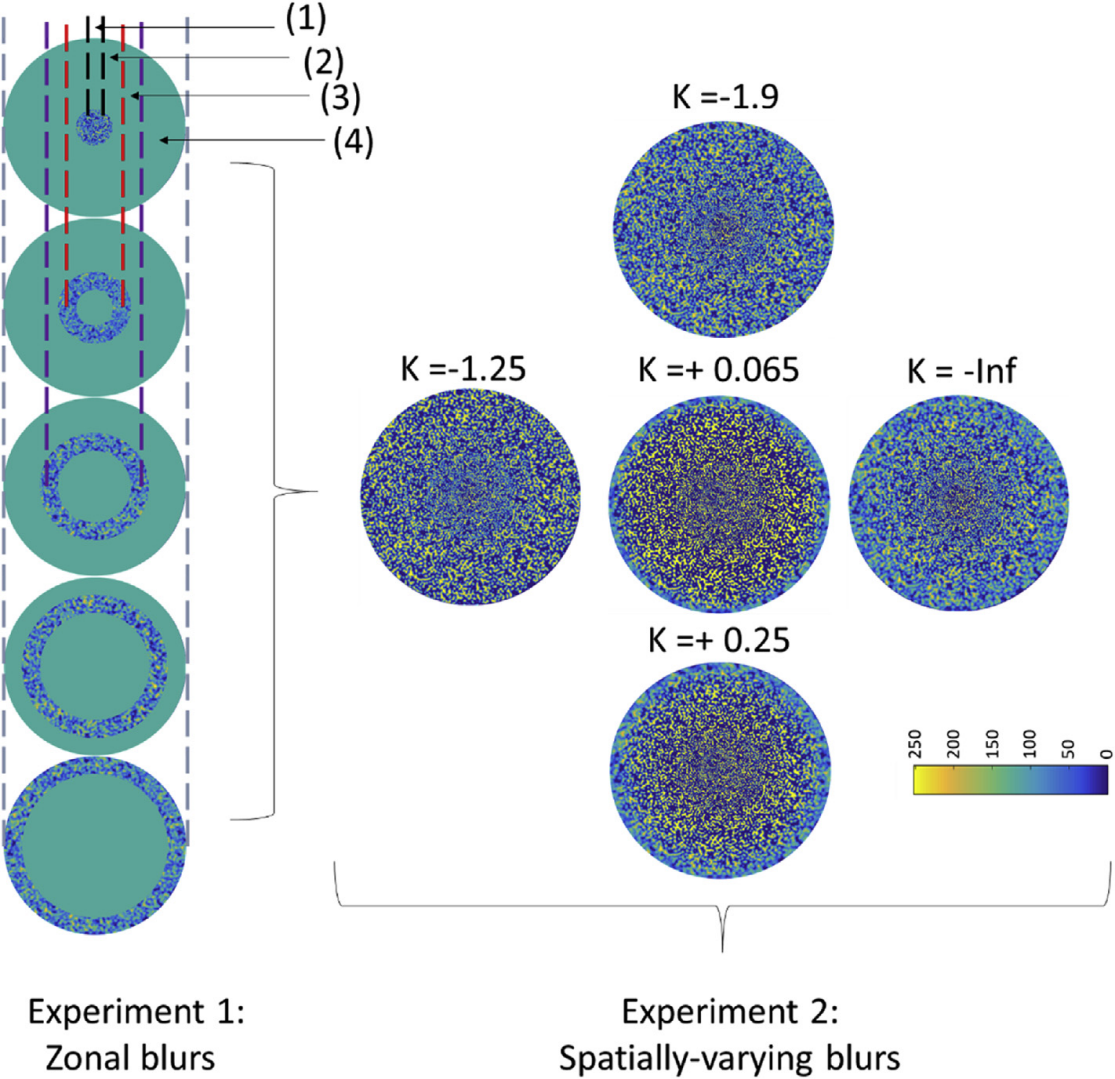
4K blurry binary noise targets projected on to the retina by the MVS, and showing the variation in blur occurring at various retinal eccentricities for the spatially-varying blur (right panel) and the zonal blur (left panel), obtained for subject S5. Each blur zone subtends a width of 1.5° and was projected in different regions of the retina, highlighted by numbers and including the foveola (1), fovea (2), parafovea (3), and perifoveal (4) regions. K indicates the slope of the blur distribution. In this example, the threshold blur distribution of the spatially-varying blur exhibited a stronger blur in the fovea (with a narrower area of high contrast, color-coded as yellow) and a smaller peripheral blur (with a finer grain) for K = -1.9, as compared to K = -Inf. Note that for subject S5, the zonal blur threshold showed lower contrast (color-coded as blue) as compared to the spatial blur, indicating enhanced threshold tolerance to blur for the zonal condition. The color of the binary noise codes for the level of contrast in the image: regions with a high concentration of yellow spot corresponds to regions of low blur having high intensity signal (value = 255) whereas regions with a high concentration of blue spot correspond to regions of high blur. The darkest blue regions correspond to regions of the binary noise having no signal.

As shown in Figure 6, the experiment comprised two tests. In the first experiment, a local blur threshold measurement (Experiment 1) was performed via a “zonal blur”. In the second experiment (Experiment 2), a global blur threshold measurement was tested via a “spatially-varying blur” binary stimulus. For both tests, the subjects participated in a training session to become familiarized with the task. The “zonal blur” binary stimulus consisted of an annulus image of about 1.5° of width displayed sequentially at five spatial locations. Consistent with the extent of the isoplanatic path of the human eye, this annulus contained only one blur level and was circumscribed by a green background. The “spatially-varying blur” consisted of an extended stimulus containing distinct levels of defocus that simulated the non-uniform retinal blur. The spatially-varying blur stimulus was split into 20 concentric rings (width = 0.375°), each displaying a single blur individually adjusted. A large number of superimposed annulus masks insured that the width of each blur level was sufficiently small to prevent noticeable, sharp blur transitions. Note that the spatially-varying blur stimulus stimulated the overall region covered by the five annuli combined together, and included the foveola, fovea, parafovea, and perifoveal regions. To simulate and study the visual effect of the increase in blur across eccentricities [20]), retinal blur variations were modelled using different sigmoid functions:

$$y=\frac{1/K\times E}{1/K-E+1}$$

where y is the normalized blur variation expressed in terms of the spherical equivalent; E is the eccentricity, and K determines the slope of the sigmoid function of the spatially-varying blur. Note that the distribution of blur $\left(y^{'}=y\times a\right)$ was assumed to be rotationally symmetric and depends only on two parameters, i.e., the K value and the defocus peak *a*, corresponding to the largest eccentricity (7.5°).

#### 2.4.6 Visual test

The tested K values (-0.8, -0.53, 1.1, 4.0, 16.7) were set to a fraction (1.5, 1.25, 0.75, 0.5, 0.25, respectively) of the total blur volume (area under the normalized sigmoid) of the linear blur *K* = 0). This range of K values was selected to cover a large range of blur variations. An interleaved staircase procedure (one-down one-up, convergence 50%) was used to control the blur levels of the binary noise. The staircase was initialized with start values, estimated in a pilot test using a stimulus with a parabolic blur distribution. The spherical equivalent M was used as a metric of perceived sharpness. During the local and global blur measurement, various annulus positions (outer eccentricities: 1.5, 3, 4.5, 6, and 7.5°) and various spatial blur profiles (slope *K*: -0.8, -0.53, 1.1, 4.0, 16.7), respectively, were randomly interleaved, and each adjustment changed the sharpness of the image by a fixed defocus amount of 0.15 D. In the global measurement, the incremental value was applied at the defocus peak, altering the set of peripheral defocus values (determined by the parameter K). For each condition, the raw data were fitted with a Gumbel function using a maximum likelihood criterion. The bothersome blur thresholds were estimated from the 63% correct point on the best-fitting Gumbel psychometric function. The standard errors of estimate were calculated with a bootstrap procedure, based on 1000 data sets simulated from the number of experimental trials at each level tested using the Palamedes toolbox [40].

## 3 Results

**Figure 7:**
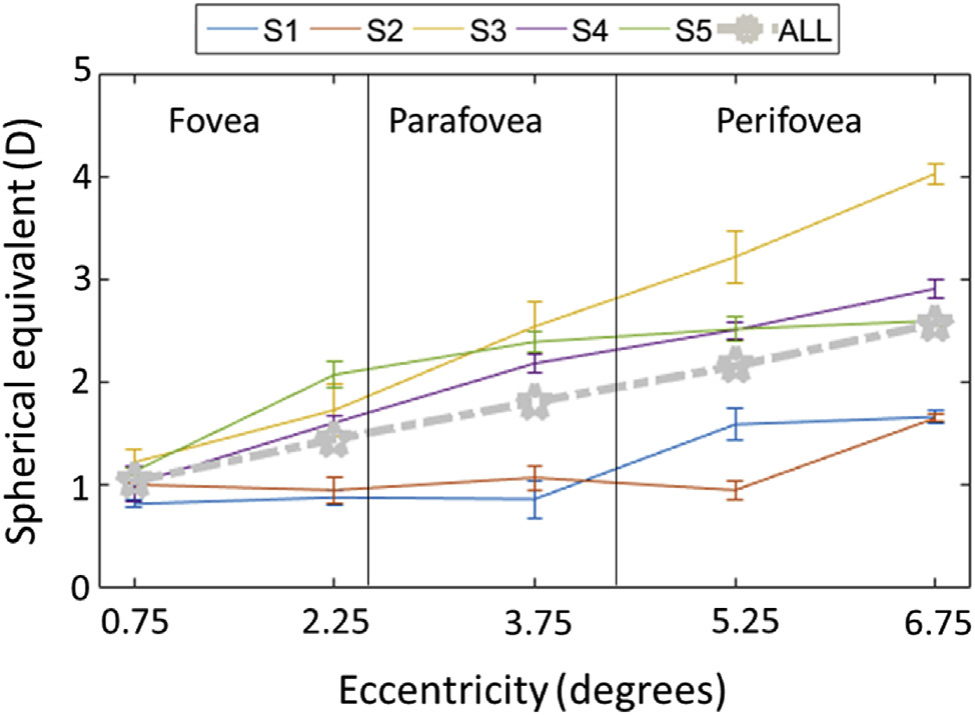
Bothersome blur threshold change for zonal blur: Bothersome blur threshold (D) measured as a function of annulus blur images centered at five eccentricities and having a field size of 1.5°. Each line represents the data of each of the five subjects. The thick grey curve represents changes in the average blur threshold of the five subjects. Error bars represent the standard errors.

As expected from previous studies, Figure 7 shows that, on average, zonal blur threshold is higher in the foveal region (E < 3°) and increases with eccentricity beyond 3°, with a slope of variation that varies across subjects. The deviation of the bothersome blur between subjects was particularly pronounced for the more peripheral regions.

**Figure 8:**
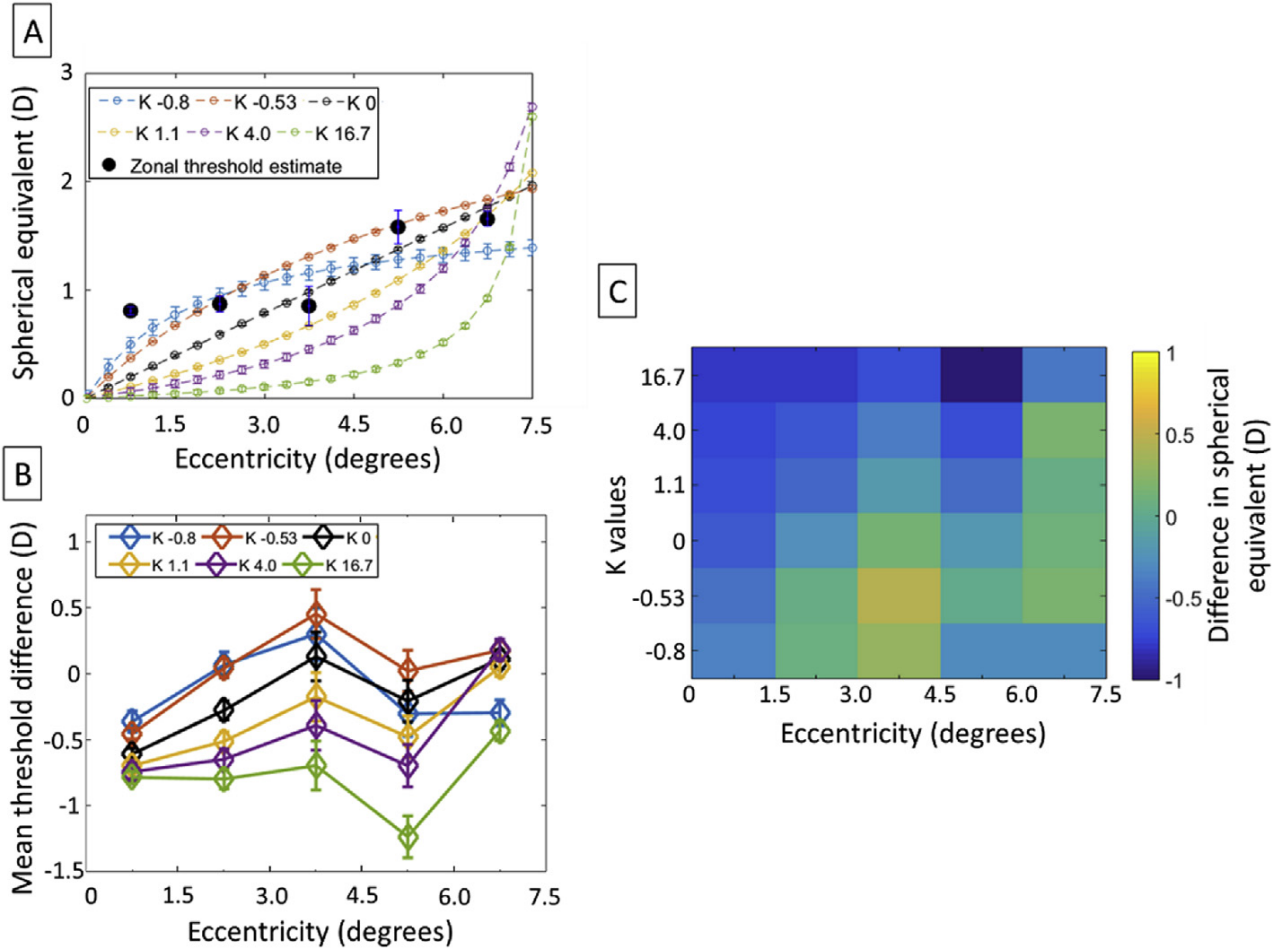
(A) Effect of blur variation for the spatially-varying blur image: Bothersome blur threshold measured in subject S1 for various sigmoid blur profiles K as a function of eccentricities. The open symbols connected with fine lines correspond to the fixed blur applied within each annular ring of the spatially-varying blur, whereas the filled symbols represent the localized bothersome blur threshold measurement shown in Figure 7. When the curve is above the filled symbol at a given eccentricity, the amount of blur tolerated at that location is higher in the presence of a compound blur. (B) Graph vector and (C) heat map plot of the corresponding threshold difference between spatially-varying blur and zonal blur over a given zonal eccentricity as a function of the K number of the sigmoid blur profile for the same subject. The calculated bothersome blur threshold of the spatially-varying blur at a given zonal eccentricity is the average blur of the threshold values under that spatial zone.

To visualize the impact of the slope of the blur distribution on the bothersome threshold, the magnitude of blur associated with each rings of the spatially varying blur (empty circle connected by a line) is plotted in Figure 8A for subject S1 as a function of K values, and compared with the local blur threshold (filled circle symbols) corresponding to the zonal blur measurement. Although the threshold profile derived from the spatially-varying blur profile at certain eccentricities was in close agreement with the local threshold obtained by measuring zonal blurs, the comparison of the zonal and global measurement shows that variation in the blur profile can modulate the impact of blur at a given eccentricity by rising or decreasing the local bothersome threshold. The difference in threshold in Figure 8A between the spatially-varying blur and zonal blur is replotted in Figure 8B by subtracting the zonal threshold and the average threshold of the spatially varying blur, which was integrated over the five rings associated with a given zonal blur.

**Figure 9:**
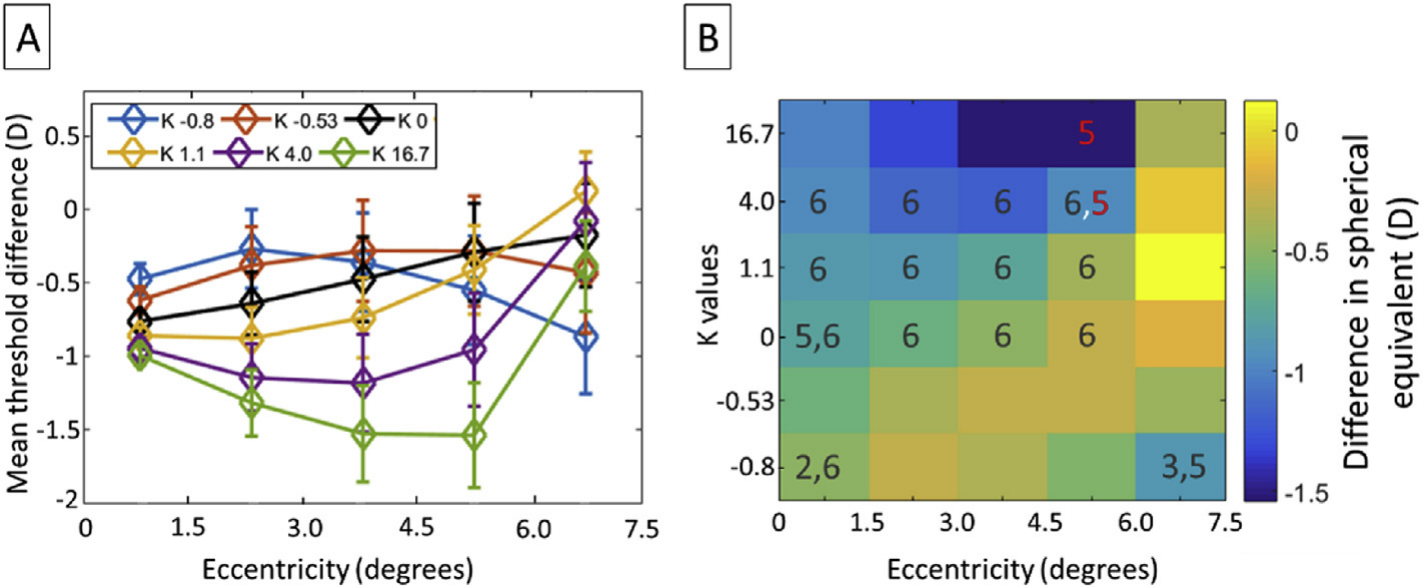
(A) Graph vector and (B) heat map plot of the estimated mean threshold differences between spatially-varying blur and zonal blur as a function of zonal eccentricity and the K number of the sigmoid blur profile for five subjects. The black and red color numbers represent the column number of K values and the row number of the eccentricities. Black color numbers indicate a statistically significant threshold difference between the blur profile of the labelled pixel and the indexed blur profile at a given eccentricity. The red numbers indicate a statistically significant blur difference between the eccentricity of the labelled pixel and the indexed eccentricity.

A two-way repeated measures analysis of variance (RANOVA) test (Eccentricity: 0.75°, 2.25°, 3.75°, 5.25°, 6.75°; K value: -0.8, -0.53, 1.1, 4.0, 16.7) on the threshold differences between zonal and spatially-varying blur was conducted using SPSS Statistics for Windows Version 25 (IBM SPSS Statistics for Windows, IBM Corporation, Armonk, NY). It revealed a significant main effect of blur distribution profile (determined by the K value) on threshold variation (F (5,20) = 14.406, p = .000,η = .783). A significant interaction was also found between the effects of eccentricity and K values (F (20,80) = 28.363, p = .000,η = .876), suggesting that the modulation of the zonal bothersome blur by blur distribution profile was affected by eccentricity. Summarized in Figure 9, the simple main effects performed with Bonferroni correction shows a differential effect of blur distribution and eccentricity on modulation of the bothersome threshold. In particular, the spatially-varying blur profile corresponding to K = 16.7 produced significantly more modulations of the bothersome blur (i.e., difference between zonal and spatially-varying blur threshold) than blur distribution with lower K values. This differential effect of blur distribution was systematic for blur with K values 1.1 and 4.0 respectively. This shows that the rate of variation of blur in the periphery matters in the perception of blur thresholds. Rapid variation in the near periphery (K = 16.7) tended to cause a larger discrepancy between zonal and global bothersome blur, with a lower blur threshold for global blur. On the contrary, at low K value and high eccentricities, the direction of modulation of the zonal bothersome by the spatially varying blur varied with subjects. We also observed a significant difference in blur threshold between the eccentricities 6.75° and 5.25° for spatially-varying blurs having a large rate of variation in the periphery K = 4.0 (mean difference = .878 ± .125, p = 0.022) and K = 16.7 (mean difference = 1.151 ± .177, p = 0.029) respectively. Overall, this indicates that the characteristic of the global blur can alter blur perception.

## 4 Discussion

This study demonstrated that it is possible to achieve a wide-angle (macula) diffraction limited image by adjustment of an optical artificial pupil, with minimal penalty on the range of normal vision (i.e., 20/20 vision). This finding extends previous investigations showing the relevance of a small pupil for simulating [41] and minimizing the visual effect of blur [42, 43, 44] on axis. While the small pupil solution is a powerful approach to flexibly test the visual effect of degradation across the macula visual field, it must be noted that it cannot fully replicate the aberrations of the human eye [45]. This is particularly true for the higher-order aberrations prevailing for large pupil size, although those fine aberrations exhibit generally a reduced visual effect off-axis. In addition, reduction of the amount of light and contrast can restrict visual assessment in free-viewing conditions using a pinhole in front of the eye. However, the present approach could provide a unique and simple solution for extending the retinal extent over which images can be considered diffraction-limited.

By testing blur perception independent of individual ocular aberrations, it was revealed that the characteristics of the blur in peripheral regions, rather than just on the blur within an isolated region (central or peripheral), can play a role in the bothersome blur threshold. More specifically, it was observed that the rate of blur change in the periphery could modulate the bothersome blur within a certain region of the retina. In the case of PALs, it is thus plausible that when looking along the corridor of the lens, the characteristics of the peripheral blur distribution (e.g., gradient of blur) in PALs can impair or increase the amount of blur that is perceived as bothersome by wearers. The measurement of peripheral blur perception in PALs may thus help to optimize lens design for personalized correction.

Minimizing unacceptable blur is imperative for the acceptance of PALs by patients. Although neurosensory measures, such as blur detection thresholds [46]), can estimate the impact of blur, not all indicate what is the maximum amount of blur induced by an ophthalmic correction that can be accepted by subjects, without invoking a sensation of annoyance or affecting performance. A distinctive aspect of the bothersome blur measure is that it does not just require a report of a change in clarity, but also some judgment about the effect of blur, as discussed by Ciuffreda et al [35]. Thus, when distributing the overall blur over the PAL surface, the bothersome blur threshold could be of relevance to determine the maximum amount of blur that can be induced in each lens region without presumably impairing performance. The relationship between foveal blur tolerance and personality shown by Woods et al [47] however suggests that an optimal choice among PALs might vary between patients, making personalized PALs potentially interesting for matching patients’ individual needs.

There are some limitations of the visual test. One of the limitations comes from the assumption that the visual system is circularly symmetric in its blur perception. However, it is shown that the visual system exhibits both optical and neural anisotropies [11], which could restrict the validity of such an assumption. It is also possible that the aberrations of the eye the visual system adapts to [48] could have influenced our measurement, and so the combined effects of blur gradient and blur asymmetry need to be accounted for. A second limitation comes from the choice of stimulus. While the use of a binary stimulus allowed fine control of the homogeneity of the image across each position of the visual field, this stimulus strongly differed by its edge structure from natural stimuli present in our environment. In particular, the falloff of the amplitude spectrum with frequency was steeper for the binary stimulus (with a slope of about -2.01) than natural visual scenes [49, 50], which have a slope of about -1. Considering that the visual system may be adapted to the “1/F” amplitude spectra of natural scenes [51], it is possible that the response of the visual system to blur for natural scenes differs for artificial stimuli and requires further investigations.

## 5 Conclusion

In conclusion, the effect of peripheral blurs on blur perception independent of individual ocular aberrations was investigated. This study showed that the surrounding blur not only alters the bothersome blur threshold, but also its complex spatial distribution. A general implication of the findings is that the visual extent over which blur matters is not limited just to the foveal central vision, suggesting that an optimal correction for central vision might not be optimal in terms of global vision, but may depend on the characteristic of the overall blur.

## Declarations

### Author contribution statement

Elie de Lestrange-Anginieur, Chea-su Kee: Conceived and designed the experiments; Performed the experiments; Analyzed and interpreted the data; Contributed reagents, materials, analysis tools or data; Wrote the paper.

### Funding statement

This work was supported by Departmental General Research Fund P0031874, School of Optometry, The Hong Kong Polytechnic University.

### Competing interest statement

The authors declare no conflict of interest.

### Additional information

No additional information is available for this paper.

## References

[1] Nowakowski M., Sheehan M., Neal D., Goncharov A.V. (2012). Investigation of the isoplanatic patch and wavefront aberration along the pupillary axis compared to the line of sight in the eye. Biomed. Optic Express.

[2] Hampson K.M. (2008). Adaptive optics and vision. J. Mod. Optic..

[3] Mathur A., Atchison D.A., Scott D.H. (2008). Ocular aberrations in the peripheral visual field. Opt. Lett..

[4] Thaung J., Knutsson P., Popovic Z., Owner-Petersen M. (2009). Dual-conjugate adaptive optics for wide-field high-resolution retinal imaging. Opt. Express.

[5] Shen J., Thibos L.N. (2011). Peripheral aberrations and image quality for contact lens correction. Optom. Vis. Sci..

[6] Meister D.J., Fisher S.W. (2008). Progress in the spectacle correction of presbyopia. Part 1: design and development of progressive lenses. Clin. Exp. Optom..

[7] Jacobs R.J., Smith G., Chan C.D. (1989). Effect of defocus on blur thresholds and on thresholds of perceived change in blur: comparison of source and observer methods. Optom. Vis. Sci..

[8] Sawides L., Dorronsoro C., Haun A.M., Peli E., Marcos S. (2013). Using pattern classification to measure adaptation to the orientation of high order aberrations. PloS One.

[9] De Lestrange-Anginieur E., Leung T.W., Kee C.S. (2020). Attentional Limit to the Resolving Power of the Eyeball.

[10] Rosen R., Lundstrom L., Unsbo P. (2011). Influence of optical defocus on peripheral vision. Invest. Ophthalmol. Vis. Sci..

[11] Zheleznyak L., Barbot A., Ghosh A., Yoon G. (2016). Optical and neural anisotropy in peripheral vision. J. Vis..

[12] Iii E.L., Campbell M.C.W., Irving E.L. (2013). Does peripheral retinal input explain the promising myopia control effects of corneal reshaping therapy (CRT or ortho-K) and multifocal soft contact lenses?. Ophthalmic Physiol. Optic..

[13] Sankaridurg P., Holden B., Smith E., Naduvilath T., Chen X., de la Jara P.L., Martinez A., Kwan J., Ho A., Frick K., Ge J. (2011). Decrease in rate of myopia progression with a contact lens designed to reduce relative peripheral hyperopia: one-year results. Invest. Ophthalmol. Vis. Sci..

[14] Wallman J., Winawer J. (2004). Homeostasis of eye growth and the question of myopia. Neuron.

[15] Troilo D., Gottlieb M.D., Wallman J. (1987). Visual deprivation causes myopia in chicks with optic-nerve section. Curr. Eye Res..

[16] Venkataraman A.P., Radhakrishnan A., Dorronsoro C., Lundstrom L., Marcos S. (2017). Role of parafovea in blur perception. Vis. Res..

[17] Webster S.M., Webster M.A., Taylor J., Jaikumar J., Verma R. (2001). Simultaneous blur contrast. Human Vis. Electr. Imag. VI.

[18] JVC Kenwood Corporation (2010). JVC Kenwood Corporation Website. https://www.jvckenwood.com/en/.

[19] Martinez-Conde S., Macknik S.L., Hubel D.H. (2004). The role of fixational eye movements in visual perception. Nat. Rev. Neurosci..

[20] Escudero-Sanz I., Navarro R. (1999). Off-axis aberrations of a wide-angle schematic eye model. J. Opt. Soc. Am. A Opt. Image Sci. Vis..

[21] Guo H., Goncharov A., Dainty C. (2012). Intraocular lens implantation position sensitivity as a function of refractive error. Ophthalmic Physiol. Optic..

[22] Sahin B., Lamory B., Levecq X., Harms F., Dainty C. (2012). Adaptive optics with pupil tracking for high resolution retinal imaging. Biomed. Optic Express.

[23] Dai G.-m. (2008). Wavefront Optics for Vision Correction.

[24] Rovamo J., Virsu V., Nasanen R. (1978). Cortical magnification factor predicts the photopic contrast sensitivity of peripheral vision. Nature.

[25] Coletta N.J., Sharma V. (1995). Effects of luminance and spatial noise on interferometric contrast sensitivity. J. Opt. Soc. Am. A Opt. Image Sci. Vis..

[26] Charman W.N., Heron G. (1988). Fluctuations in accommodation: a review. Ophthalmic Physiol. Optic..

[27] Vinas M., Dorronsoro C., Gonzalez V., Cortes D., Radhakrishnan A., S Marcos S. (2017). Testing vision with angular and radial multifocal designs using Adaptive Optics. Vis. Res..

[28] Chen L., Artal P., Gutierrez D., Williams D.R. (2007). Neural compensation for the best aberration correction. J. Vis..

[29] Artal P., Chen L., Fernández E.J., Singer B., Manzanera S., Williams D.R. (2004). Neural compensation for the eye’s optical aberrations. J. Vis..

[30] Chen L., Singer B., Guirao A., Porter J., Williams D.R. (2005). Image metrics for predicting subjective image quality. Optom. Vis. Sci..

[31] Rovamo J., Virsu V. (1979). An estimation and application of the human cortical magnification factor. Exp. Brain Res..

[32] Galvin S.J., O'Shea R.P., Squire A.M., Govan D.G. (1997). Sharpness overconstancy in peripheral vision. Vis. Res..

[33] Atchison D.A., Fisher S.W., Pedersen C.A., Ridall P.G. (2005). Noticeable, troublesome and objectionable limits of blur. Vis. Res..

[34] Atchison D.A., Guo H., Charman W.N., Fisher S.W. (2009). Blur limits for defocus, astigmatism and trefoil. Vis. Res..

[35] Ciuffreda K.J., Selenow A., Wang B., Vasudevan B., Zikos G., Ali S.R. (2006). Bothersome blur": a functional unit of blur perception. Vis. Res..

[36] Goodman J.W. (1996). Introduction to Fourier Optics.

[37] Thibos L.N., Applegate R.A., Schwiegerling J.T., Webb R., Members V.S.T. (2002). Standards for reporting the optical aberrations of eyes. J. Refract. Surg..

[38] Thibos L.N., Hong X., Bradley A., Cheng X. (2002). Statistical variation of aberration structure and image quality in a normal population of healthy eyes. J. Opt. Soc. Am. A.

[39] Brainard D.H. (1997). The psychophysics toolbox. Spatial Vis..

[40] Prins N., Kingdom F.A.A. (2009). Palamedes: Matlab Routines for Analyzing Psychophysical Data.

[41] Jacobs R.J., Bailey I.L., Bullimore M.A. (1992). Artificial pupils and maxwellian view. Appl. Optic..

[42] Atchison D.A., Smith G., Efron N. (1979). The effect of pupil size on visual acuity in uncorrected and corrected myopia. Am. J. Optom. Physiol. Opt..

[43] Xu R., Thibos L., Bradley A. (2016). Effect of target luminance on optimum pupil diameter for presbyopic eyes. Optom. Vis. Sci..

[44] Xu R., Gil D., Dibas M., Hare W., Bradley A. (2016). The effect of light level and small pupils on presbyopic reading performance. Invest. Ophthalmol. Vis. Sci..

[45] Ohlendorf A., Tabernero J., Schaeffel F. (2011). Visual acuity with simulated and real astigmatic defocus. Optom. Vis. Sci..

[46] Atchison D.A., Charman W.N., Woods R.L. (1997). Subjective depth of focus of the eye. Optom. Vis. Sci..

[47] Woods R.L., Colvin C.R., Vera-Diaz F.A., Peli E. (2010). A relationship between tolerance of blur and personality. Invest. Ophthalmol. Vis. Sci..

[48] Sawides L., de Gracia P., Dorronsoro C., Webster M.A., Marcos S. (2011). Vision is adapted to the natural level of blur present in the retinal image. PloS One.

[49] Tolhurst D.J., Tadmor Y., Chao T. (1992). Amplitude spectra of natural images. Ophthalmic Physiol. Optic..

[50] Field D.J., Brady N. (1997). Visual sensitivity, blur and the sources of variability in the amplitude spectra of natural scenes. Vis. Res..

[51] Webster M.A., Miyahara E. (1997). Contrast adaptation and the spatial structure of natural images. J. Opt. Soc. Am. A.

